# The whole-genome sequence and assembly of *Alternaria alternata* isolate Karbala-1, causative agent of black point in wheat

**DOI:** 10.1128/mra.00283-26

**Published:** 2026-06-22

**Authors:** Zainab L. Hameed, Ban T. Mohammed, Zuhair M. Jeddoa, Adnan A. Lahuf

**Affiliations:** 1 Field Crops Department, Agriculture College, University of Kerbala 125663, Karbala, Iraq; 2 Department of Biology, College of Education for Pure Science, University of Kerbala 566625, Karbala, Iraq; 3 College of Pharmacy, Al-Zahraa University for Women 608869, Karbala, Iraq; 4 Plant Protection Department, Agriculture College, University of Kerbala 125663, Karbala, Iraq; University of California Riverside, Riverside, California, USA

**Keywords:** *Alternaria alternata*, genome, black point, pathogenic isolate, Iraq

## Abstract

In this study, we report the draft genome sequence of *Alternaria alternata* recovered from wheat seeds exhibiting black point symptoms from Karbala Province, Iraq. The assembled genome of *A. alternata* isolate Karbala-1 has an estimated size of 34.99 Mb with a GC content of 50.98%.

## ANNOUNCEMENT

The filamentous ascomycete fungus *Alternaria alternata* is an important toxigenic species with a multinational distribution (1). It occurs on a wide range of natural substrates, where it can function as a saprophyte or endophyte. In addition, *A. alternata* is a significant plant pathogen capable of infecting numerous host plants worldwide, including those cultivated in Iraq (2–4).

Previous field surveys conducted in 2024–2025 in major wheat-growing regions of Karbala Province, Iraq (43°49′11″, 32°30′30), revealed the widespread occurrence of wheat grains affected by black point disease across all surveyed locations and commonly cultivated wheat genotypes. Among the associated fungal species, *Alternaria alternata* was isolated from symptomatic grain samples that were surface-sterilized with 2% sodium hypochlorite solution for 3 min, rinsed thoroughly with distilled water, and subsequently disinfected with 70% ethanol for 30 s before being dried on sterile filter paper and plated on sold water agar (WA) media, and incubated at 25°C ± 2°C for 3 days. Emerging fungal colonies were then transferred onto potato dextrose agar (PDA) plates for purification (5).

The fungus was then identified as the predominant pathogen. The species identification was initially based on morphological characteristics and subsequently confirmed through multilocus molecular characterization using Sanger sequencing of four genetic markers (6): the internal transcribed spacer (ITS), translation elongation factor 1-alpha (TEF1-α), β-tubulin, and RNA polymerase II largest subunit (RPB1) (7–9).

To confirm species identity, the query sequences of these loci were compared against the NCBI non-redundant nucleotide (nt) database using BLASTn analysis (10) that showed high sequence similarity (98%–99%) with previously reported *A. alternata* isolates, supporting the taxonomic assignment of the recovered isolate.

Following single-spore isolation and purification on solid PDA media, the confirmed *Alternaria alternata* isolate Karbala-1 was cultured for 7 days under laboratory conditions. Genomic DNA was subsequently extracted and the sample was submitted to BGI (Hong Kong, China) for whole-genome sequencing. Genomic DNA was extracted using a magnetic bead-based method, followed by magnetic separation, washing, and elution of purified DNA for downstream molecular analyses (11), after which sequencing libraries were constructed using the MGIEasy Universal DNA Library Prep Set (MGI). The prepared libraries were sequenced on the DNBSEQ-T7 platform (BGI) utilizing DNA nanoball (DNB) technology to generate 150-bp paired-end reads.

A total of 8,183,273 raw paired-end reads were generated for *A. alternata* isolate Karbala-1. The raw sequencing reads were subjected to quality control and filtering to remove adapter sequences, low-quality bases, short reads, and PCR duplicates using SOAPnuke (version 2.X) with the following parameters: -n 0.01 -l 20 -q 0.4 --adaMis 3 --rmdup --minReadLen 150 (12). The resulting high-quality reads were assembled *de novo* using SPAdes version 4.0.0 (13). The assembled contigs were subsequently scaffolded in Geneious Prime v2025.2.1 using the reference genome of *Alternaria alternata* (RefSeq accession GCF_001642055.1). Contigs and scaffolds shorter than 500 bp were excluded from the final draft genome assembly.

The final genome assembly comprised 126 scaffolds (>1,000 bp) with a total length of 34,990,736 bp (34.9 Mb). The assembly showed a scaffold N50 of 2,814,812 bp and an L50 of 5. The longest scaffold measured 6,787,648 bp, and the overall GC content of the genome was 50.98%. The estimated sequencing coverage reached 716.155×. The draft genome sequence of *Alternaria alternata* isolate Karbala-1 has been deposited in the NCBI database.

## Data Availability

The genome data generated in this study are publicly available. The BioProject accession number is PRJNA1187237. The BioSample accession number for the isolate, Karbala-1, is SAMN44786738. The Whole-Genome Shotgun projects for this isolate have been deposited at DDBJ/ENA/GenBank under the accession number JBKAZY000000000. The raw data have been deposited in the NCBI Sequence Read Archive (SRA) SRR37518749. The obtained sequences were deposited in GenBank under accession numbers PQ217832.1, PQ212710.1, PQ212709.1, and PQ212708.1, respectively. All records can be accessed through the NCBI database at https://www.ncbi.nlm.nih.gov.
